# Cytostatic and Pro-Apoptotic Effects of *Justicia spicigera* Schltdl. on LNCaP Prostate Cancer Cells: Role of G_0_/G_1_ Cell Cycle Arrest and Phytochemical Characterization

**DOI:** 10.3390/plants15060944

**Published:** 2026-03-19

**Authors:** Ivette Bravo-Espinoza, Fabiola Hernández Rosas, Maria Elena Hernández-Aguilar, Marycarmen Godínez-Victoria, Rodrigo Rafael Ramos-Hernández, Carlos Alberto López-Rosas, Santiago González-Periañez, Ezri Cruz-Pérez, Fernando Rafael Ramos-Morales, Tushar Janardan Pawar

**Affiliations:** 1 Facultad de Química Farmacéutica Biológica, Universidad Veracruzana, Circuito Gonzalo Aguirre Beltrán S/N, Zona Universitaria, Xalapa 91090, Mexico; ivbravo@uv.mx (I.B.-E.); carloslopez02@uv.mx (C.A.L.-R.); ezcruz@uv.mx (E.C.-P.); 2 Instituto de Investigaciones Cerebrales, Universidad Veracruzana, Luis Castelazo Ayala S/N, Col. Industrial Animas, Xalapa 91190, Mexico; elenahernandez@uv.mx; 3 Centro de Investigación, Universidad Anáhuac Querétaro, Queretaro Circuito Universidades I, Fracción 2 S/N, Zibatá, El Marqués 76246, Mexico; fabiola.hernandezro@anahuac.mx; 4 Facultad de Química, Universidad Autónoma de Querétaro, Querétaro 76010, Mexico; 5 Sección de Estudios de Posgrado e Investigación, Escuela Superior de Medicina, Instituto Politécnico Nacional, Mexico City 07738, Mexico; mgodinez@ipn.mx; 6 Instituto de Química Aplicada, Universidad Veracruzana, Luis Castelazo Ayala s/n, Col. Industrial Animas, Xalapa 91190, Mexico; rodrigoqfb@gmail.com (R.R.R.-H.); santiagonzalez@uv.mx (S.G.-P.); 7 Dirección de Mecatrónica, Universidad Politécnica de Querétaro, Carretera Estatal 420 S/N, El Rosario 76240, Mexico

**Keywords:** *Justicia spicigera*, muicle, LNCaP cells, prostate cancer, kaempferitrin, apoptosis, cell cycle arrest, Mexican ethnomedicine

## Abstract

*Justicia spicigera* is a central medicinal plant in Mexican ethnomedicine, yet its therapeutic potential against prostate cancer remains largely unexplored. This study investigated the antiproliferative and pro-apoptotic effects of a 50% hydroalcoholic extract from the leaves and stems of *J. spicigera* on androgen-sensitive LNCaP prostate cancer cells. Phytochemical profiling via thin layer chromatography (TLC) and LC-MS putatively identified the bioactive flavonoid kaempferitrin within the complex extract. Biological assays, including MTT, trypan blue exclusion, and flow cytometry, revealed that the extract inhibits LNCaP proliferation in a distinct, dose-dependent manner. At a lower concentration (250 µg/mL), the extract exerted a primarily cytostatic effect by inducing significant G_0_/G_1_ cell cycle arrest without triggering immediate cell death. Conversely, higher concentrations (≥500 µg/mL) were potently cytotoxic, reducing cell viability to below 20% and inducing late apoptosis in approximately 58% of the population within 24 h. These results validate the in vitro biological activity of *J. spicigera* in a specific prostate cancer model. While the concentrations required for cytotoxicity are relatively high, the observed dose-dependent G_0_/G_1_ arrest provides a foundational phenotypic profile for this traditional extract, warranting further investigation into its selectivity and potential as a bioactive scaffold.

## 1. Introduction

Prostate cancer (PCa) is one of the most frequently diagnosed malignancies in men and remains a primary driver of cancer-related mortality globally [1,2]. Along with benign prostatic hyperplasia (BPH), these conditions represent a substantial burden for patients and healthcare systems, particularly in Mexico. In 2023, Mexico recorded 7241 deaths from prostate cancer, reflecting a mortality rate of 34.6 per 100,000 men over the age of 40. For 2024, the incidence rate was reported at 28.9 per 100,000 men in the same age group, totaling 6050 new cases. The prevalence of non-malignant disease is even more pronounced; BPH accounted for 123,215 new cases in 2024, an incidence rate of 589.4 per 100,000 men. PCa is a highly heterogeneous disease that progresses through localized, advanced, and metastatic stages, each requiring specific therapeutic interventions [3,4,5]. While environmental factors and genetic predisposition influence onset, mutations in genes such as BRCA1, BRCA2, and TP53, which regulate DNA repair and androgen metabolism, significantly contribute to disease progression [6].

The androgen receptor (AR) signaling pathway is the central axis of PCa biology and a primary therapeutic target. Conventional strategies, including androgen deprivation therapy (ADT), chemotherapy, and immunotherapy, have shown clinical efficacy; however, the development of drug resistance and treatment-associated toxicities underscores the urgent need for alternative or complementary approaches [7]. Historically, traditional medicine has been a cornerstone of cancer therapy, with medicinal plants serving as essential sources of bioactive compounds for drug discovery. In Mexico, species such as ‘cuachalalate’ (*Amphipterygium adstringens*) are used for inflammatory conditions, while soursop (*Annona muricata*) is utilized for its reputed antitumor properties [8,9]. Additionally, species of the genus *Verbesina* have gained scientific attention for their bioactive secondary metabolites and traditional use in treating neoplastic conditions, further illustrating the therapeutic potential of the Mexican flora [10,11].

The importance of natural products in modern oncology is further highlighted by the fact that several FDA-approved agents, including paclitaxel, vinblastine, and camptothecin, are derived from these sources [12]. These limitations underscore the need for alternative strategies, including the use of medicinal plants that offer low toxicity and broader accessibility. *Justicia spicigera* Schltdl., commonly known as “muicle”, a plant widely used in Mexican ethnomedicine, has attracted scientific interest. In Mexican ethnomedicine, it has been widely utilized for treating anemia, menstrual irregularities, respiratory diseases, and microbial infections [13,14,15,16,17]. Its diverse bioactive compounds, primarily from the leaves and aerial parts, including various flavonoids and anthocyanins, are known to exhibit anticancer, anti-inflammatory, and immunomodulatory properties [18,19,20]. However, despite its broad therapeutic applications, research on the potential anticancer effects of *J. spicigera* remains limited. Several studies have reported the presence of flavonoids, phytosterols, and terpenoids in *J. spicigera*, many of which exhibit promising anticancer properties [21,22,23,24]. Kaempferitrin and quercetin derivatives, found in *J. spicigera*, have demonstrated antiproliferative and apoptotic activity, acting through the modulation of p53, Bcl-2, Bax, and cyclins, which regulate cell cycle progression and programmed cell death [25]. Additionally, β-sitosterol, a major phytosterol in *J. spicigera*, has been shown to inhibit prostate cancer cell proliferation by modulating ceramide metabolism and AR signaling [26].

Although the cytotoxic effects of an extract from the leaves of *J. spicigera* have been reported in leukemia (TF-1), cervical (HeLa), and breast (T47D) cancer cells [27,28], its potential cytotoxic effect in prostate cancer, particularly in androgen-sensitive models like LNCaP, remains largely unexplored. Previous studies have demonstrated that an extract from the leaves of *J. spicigera* induces apoptosis at low micromolar concentrations, with IC_50_ values as low as 17 μg/mL in HeLa cells [29]. Additionally, an in vivo study showed that *J. spicigera* extract suppressed tumor growth by up to 53%, comparable to the effects of cisplatin, suggesting significant anticancer potential [30].

One of the major challenges in prostate cancer therapy is overcoming drug resistance and systemic toxicity associated with conventional treatments [7]. Many standard chemotherapeutics exert their effects by inducing direct cytotoxicity, leading to significant side effects and the selection of resistant cancer cell populations. In contrast, cytostatic agents, which inhibit cell proliferation without immediately triggering apoptosis, offer an alternative approach that may delay tumor progression and reduce therapy resistance [31].

Given the growing interest in plant-derived cytostatic compounds, it is critical to investigate whether *J. spicigera* primarily acts via cell cycle modulation or through direct apoptosis induction. The presence of flavonoids and sterols in *J. spicigera* suggests a possible mechanism involving G_0_/G_1_ arrest and subsequent apoptosis induction, as previously demonstrated in other plant-derived anticancer compounds [30]. However, no study has comprehensively evaluated these effects in prostate cancer models, warranting further investigation.

Despite preliminary evidence of anticancer activity, studies assessing *J. spicigera* in prostate cancer models, especially in androgen-responsive LNCaP cells, remain scarce. A prior study reported that a hydroalcoholic extract from the aerial parts of *J. spicigera* induced a modest G_0_-phase cell cycle arrest in LNCaP cells at high concentrations (IC_50_ = 3026 ± 421 µg/mL) but lacked significant apoptotic effects. The limited apoptotic response observed in earlier studies prompts a closer examination of whether *J. spicigera* can activate programmed cell death pathways at lower, therapeutically relevant concentrations, an aspect that has not been thoroughly addressed to date. This study presents a prospective investigation into the concentration-dependent activity of *J. spicigera* on LNCaP cells. While a prior study characterized this species as purely cytostatic (IC_50_ > 4000 µg/mL) [32], we hypothesize that specific concentration thresholds (≥500 µg/mL) can trigger a transition from G_0_/G_1_ arrest to programmed cell death (apoptosis). Kaempferitrin was selected as the marker of interest due to its structural potential to influence steroid-signaling pathways, providing a mechanistic framework for exploring the ‘muicle’ extract in an androgen-sensitive context, determining its dose-dependent cytostatic and cytotoxic activity, and elucidating its impact on cell cycle progression and apoptosis.

## 2. Results

### 2.1. Phytochemical Profiling of J. spicigera Extract

**Figure 1:**
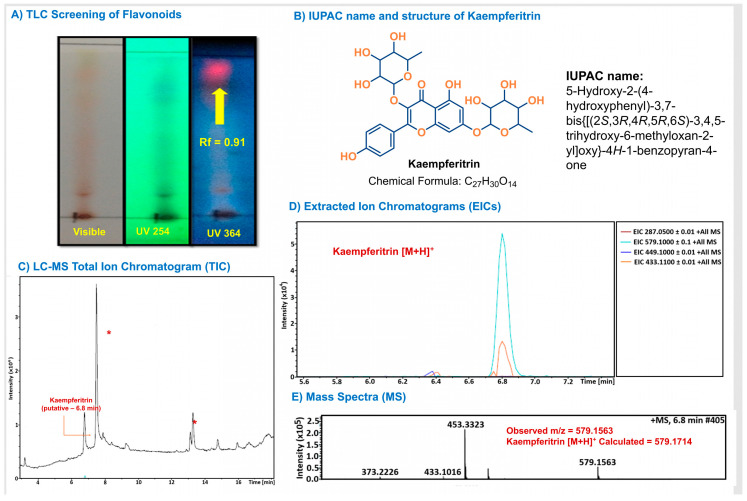
Preliminary phytochemical characterization of the hydroalcoholic extract of *J. spicigera* leaves and stems. (**A**) Preliminary TLC analysis developed with 1% aluminum chloride and visualized under UV 365 nm light. The arrow indicates a spot (Rf = 0.91) with fluorescence characteristic of the flavonoid class. (**B**) Structure and IUPAC name of kaempferitrin (**C**) LC-MS Total Ion Chromatogram (TIC) Major, unidentified components are marked with an asterisk (*). (**D**) Extracted Ion Chromatograms (EICs) for key masses. (**E**) Mass spectrum from the peak at 6.8 min.

The phytochemical composition of the *J. spicigera* extract was investigated using chromatographic and mass spectrometric techniques, as summarized in Figure 1. The analysis suggests the presence of the flavonoid kaempferitrin (Figure 1B) [18]. Preliminary screening by analytical thin-layer chromatography (TLC) indicated the presence of flavonoids in the extract. When developed with a flavonoid-specific reagent, a prominent spot was observed with strong fluorescence under UV 365 nm light (Figure 1A) [22,30].

To further investigate the composition, the extract was analyzed by Liquid Chromatography-Mass Spectrometry (LC-MS). The Total Ion Chromatogram (TIC) revealed a complex mixture containing multiple components, with major, unidentified compounds eluting at approximately 7.5 min and 13.3 min (Figure 1C, marked with asterisks). Based on literature reports of flavonoids in *J. spicigera*, the data was searched for the mass corresponding to kaempferitrin. The Extracted Ion Chromatogram (EIC) for its protonated molecule ([M + H]^+^, *m*/*z* 579.1) showed a distinct peak at a retention time of approximately 6.8 min (Figure 1D). The mass spectrum of this peak confirmed an ion at *m*/*z* 579.1563. The mass spectrum of this peak confirmed an ion at *m*/*z* 579.1563. This observed value represents a mass error of 26.1 ppm compared to the calculated exact mass for the kaempferitrin [M + H]^+^ ion (579.1714), supporting its putative identification. Furthermore, high-resolution analysis of the major unidentified peaks at 7.5 and 13.3 min (Figure 1C) provides a rigorous chemical fingerprint that distinguishes this specific hydroalcoholic batch from previously reported extracts. This observed mass corresponds closely to the calculated exact mass for the kaempferitrin [M + H]^+^ ion (579.1714), supporting its putative identification in the extract (Figure 1E) [25].

The presence of multiple fluorescent spots in the TLC analysis (Figure 1A) and prominent peaks in the TIC (Figure 1C) underscores the phytochemical complexity of the *J. spicigera* hydroalcoholic extract. Although the TIC reveals major unidentified peaks at approximately 7.5 and 13.3 min, kaempferitrin was selected as the analytical marker for this study due to its established role in the literature as a key bioactive flavonoid in this species. The identification of this compound, even as a minor component relative to the total extract, aligns with the traditional use of this plant in Mexican ethnomedicine for treating neoplastic conditions. Furthermore, in accordance with the principles of ethnopharmacology, the evaluation of the whole extract is essential to capture potential synergistic or additive interactions between these diverse constituents, which are often lost upon the isolation of individual monomeric compounds.

### 2.2. Effects of J. spicigera on LNCaP Cell Proliferation

To investigate the antiproliferative effects of *J. spicigera* extract, a combination of colorimetric and microscopic assays was used to assess cell viability, morphology, and membrane integrity. The results demonstrated a dose- and time-dependent inhibition of LNCaP cell proliferation, with evidence of cytostatic effects at sub-lethal concentrations and cytotoxic responses at higher doses.

#### 2.2.1. MTT Assay

**Figure 2:**
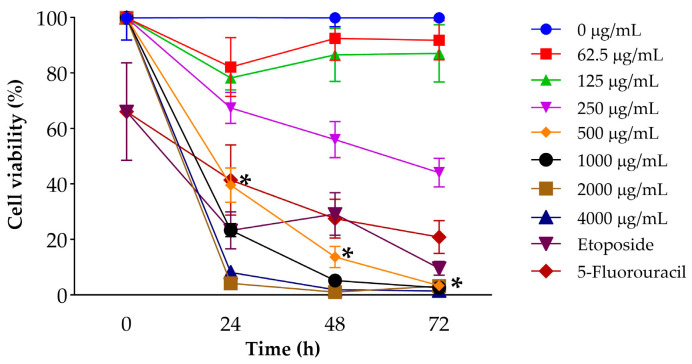
Effect of *J. spicigera* extract on LNCaP cell viability. Cells were treated with increasing concentrations of the extract for 24, 48, and 72 h. Cell viability was assessed by the MTT assay and is expressed as a percentage relative to the untreated control (0 µg/mL), which is set at 100%. Positive controls include 10 µM etoposide and 10 µM 5-fluorouracil. Values are presented as mean ± SEM (*n* = 9). * *p* < 0.001 compared to the untreated control.

The metabolic activity of LNCaP cells was evaluated using the MTT assay after exposure to increasing concentrations of *J. spicigera* extract (62.5–4000 µg/mL) for 24, 48, and 72 h. At 250 µg/mL, no significant reduction in viability was observed at 24 h, suggesting minimal cytotoxicity at this concentration. However, at 500 µg/mL, a marked reduction in cell viability was detected (*p* < 0.001), which became more pronounced at extended time points. At higher concentrations (1000–4000 µg/mL), viability decreased to below 20% by 72 h, closely resembling the effect of the positive control etoposide (10 µM) (Figure 2).

#### 2.2.2. Morphological Characterization of Treated LNCaP Cells

**Figure 3:**
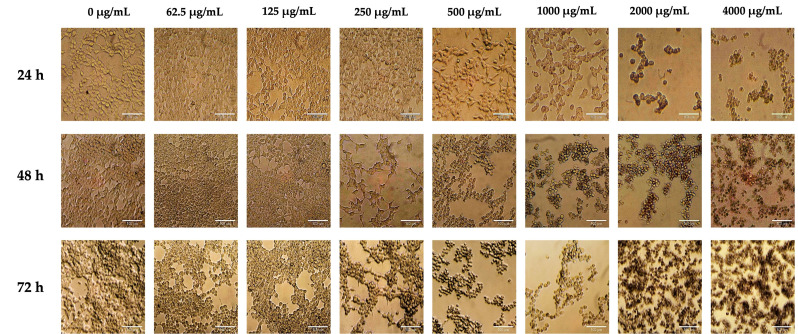
Morphological analysis of LNCaP cells treated with *J. spicigera* extract at 250 and 500 µg/mL for 24 h, 48 h and 72 h. Representative phase-contrast micrographs demonstrate dose- and time-dependent morphological changes, including cell rounding, detachment, and reduced adherence. Scale bar = 100 µm.

To complement the metabolic viability data, phase-contrast microscopy was performed to assess the morphological changes induced by the extract. Untreated control cells maintained a polygonal epithelial morphology with dense, adherent colonies typical of LNCaP cells. Following treatment with 250 µg/mL extract, cells exhibited slight reductions in colony density and spreading at 48 h, without evidence of membrane disruption or blebbing, findings consistent with a cytostatic response. In contrast, cells exposed to 500 µg/mL showed early signs of apoptosis as early as 24 h, including cell shrinkage, rounding, and partial detachment from the substrate. These changes intensified at 48 h, marked by pronounced membrane blebbing and disruption of the monolayer (Figure 3).

#### 2.2.3. Trypan Blue Assay

**Figure 4:**
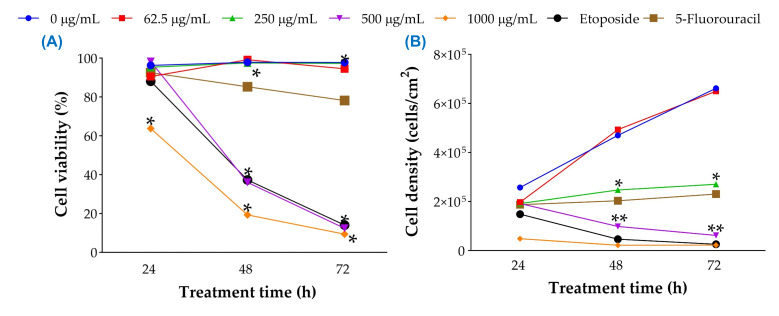
Trypan blue exclusion assay results showing viable and non-viable LNCaP cells after treatment with *J. spicigera* extract at 24, 48, and 72 h. Positive controls (10 µM etoposide and 10 µM 5-fluorouracil) were included for comparison, (**A**) Cell viability and (**B**) Cell density. Values represent mean ± SEM (n = 9). Statistical significance: (* *p* < 0.05, ** *p* < 0.001).

To directly assess membrane integrity and distinguish viable from non-viable cells, the trypan blue exclusion assay was conducted under the same treatment conditions. At 250 µg/mL, no significant reduction in viable cell counts was observed at 24 h, while a modest but significant decline emerged by 48 h (*p* < 0.05), consistent with delayed cytostatic effects. At 500 µg/mL, viability dropped sharply at 48 and 72 h (*p* < 0.001), corroborating the cytotoxic threshold observed in MTT (Figure 4A) and morphological analyses (Figure 4B).

### 2.3. Apoptosis Induction and Cell Viability

**Figure 5:**
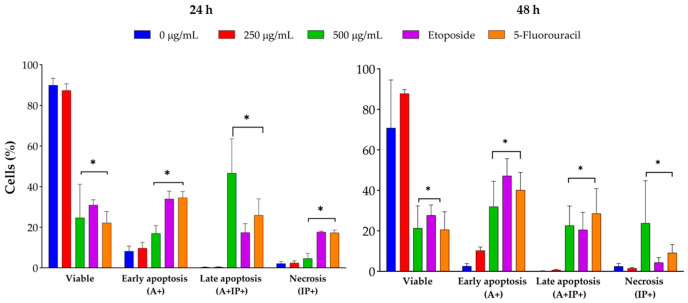
Flow cytometry analysis of LNCaP cells treated with *J. spicigera* extract at 250, 500, and 1000 µg/mL for 24 and 48 h. Apoptotic cells were detected using Annexin V/PI staining. Positive controls include 10 µM etoposide and 10 µM 5-fluorouracil. Bars represent mean ± SEM (n = 3). (* *p* < 0.001).

*J. spicigera* extract induced apoptosis in LNCaP cells in a dose-dependent manner, as determined by Annexin V/PI staining and flow cytometry analysis (Figure 5). At 250 µg/mL, the percentage of apoptotic cells remained comparable to that of untreated controls, confirming that this concentration primarily exerts a cytostatic rather than cytotoxic effect. However, at 500 µg/mL, a significant increase in early apoptosis was observed at 24 h, progressing to late apoptosis at 48 h (*p* < 0.001 vs. control). At concentrations of 1000 µg/mL and above, apoptosis was predominant, with a concurrent increase in necrotic cell death, similar to the effect observed with etoposide (positive control).

**Figure 6:**
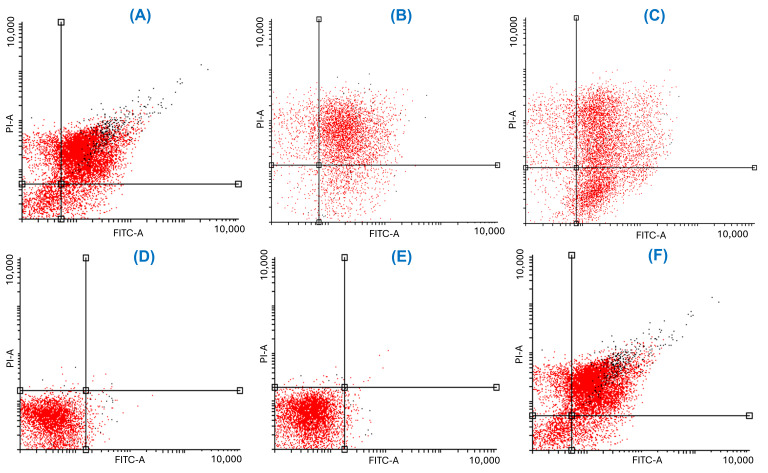
Annexin V-FITC/PI flow cytometry dot plots of LNCaP cells at 24 h. Treatments include: (**A**) negative control (untreated), and positive controls (**B**) 10 µM etoposide and (**C**) 10 µM 5-fluorouracil); and *J. spicigera* extract at concentrations of (**D**) 62.5 µg/mL, (**E**) 250 µg/mL, (**F**) 500 µg/mL. Individual cellular events are represented by dots, where the color gradient from black to red indicates increasing cell density within a specific population. The horizontal and vertical lines represent quadrant gates (thresholds) established based on the negative control to distinguish between viable (lower-left: Annexin V−/PI−), early apoptotic (lower-right: Annexin V+/PI−), late apoptotic (upper-right: Annexin V+/PI+), and necrotic (upper-left: Annexin V−/PI+) cell populations. Apoptotic populations shift toward the lower-right and upper-right quadrants in a dose-dependent manner.

**Figure 7:**
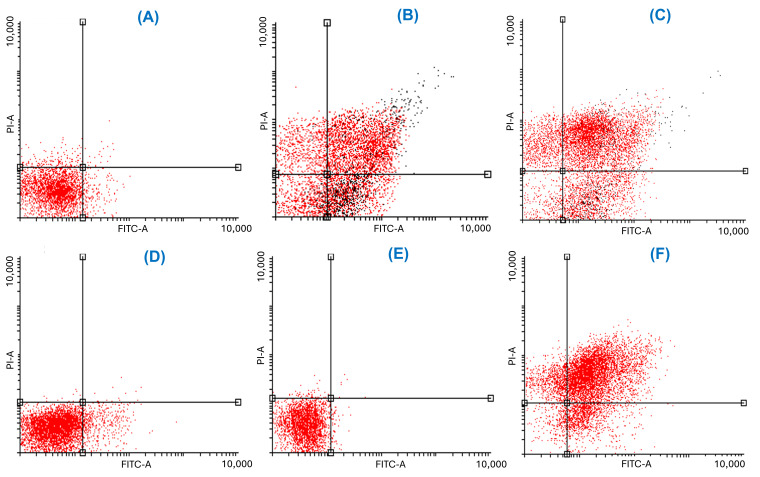
Annexin V-FITC/PI flow cytometry dot plots of LNCaP cells at 48 h. Treatments include: (**A**) negative control (untreated), and positive controls (**B**) 10 µM etoposide and (**C**) 10 µM 5-fluorouracil); and *J. spicigera* extract at concentrations of (**D**) 62.5 µg/mL, (**E**) 250 µg/mL, (**F**) 500 µg/mL. Individual cellular events are represented by dots, where the color gradient from black to red indicates increasing cell density within a specific population. The horizontal and vertical lines represent quadrant gates (thresholds) established based on the negative control to distinguish between viable (lower-left: Annexin V−/PI−), early apoptotic (lower-right: Annexin V+/PI−), late apoptotic (upper-right: Annexin V+/PI+), and necrotic (upper-left: Annexin V−/PI+) cell populations. Apoptotic populations shift toward the lower-right and upper-right quadrants in a dose-dependent manner.ile.

The dot plots showing Annexin V/PI staining at 24 and 48 h provide visual confirmation of the dose-dependent shift from viability to apoptosis (Figure 6 and Figure 7). At 24 h, a shift from viable to apoptotic quadrants was evident at ≥500 µg/mL. At 48 h, this shift was more pronounced, with the highest concentrations inducing widespread late apoptosis and necrosis, while 250 µg/mL maintained a largely viable profile.

**Figure 8:**
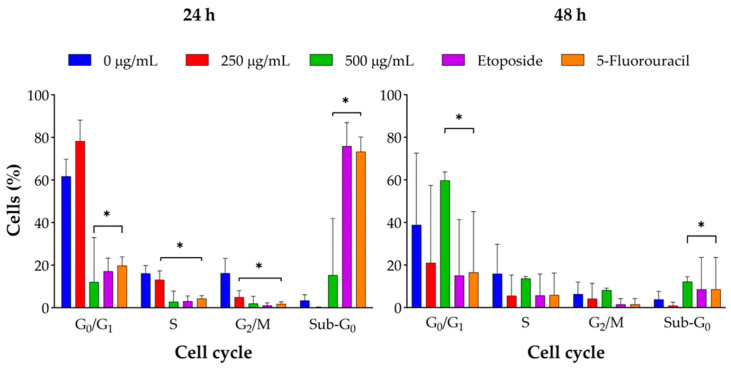
*J. spicigera* extract induces G_0_/G_1_ cell cycle arrest in LNCaP cells. Cells were treated with the indicated concentrations for 24 h (**left panel**) and 48 h (**right panel**). Positive controls consist of 10 µM etoposide and 10 µM 5-fluorouracil. Cell cycle distribution was determined by propidium iodide (PI) staining and flow cytometry. The graphs show the percentage of cells in the G_0_/G_1_, S, G_2_/M, and Sub-G_0_ phases. Values are presented as mean ± SEM (n = 3). * *p* < 0.001 compared to the corresponding control group.

To further distinguish between viable and non-viable cells, flow cytometry with propidium iodide (PI) exclusion was conducted (Figure 8). At 250 µg/mL, the percentage of viable cells remained above 85% at both 24 and 48 h. In contrast, at 500 µg/mL, a significant reduction in viable cell populations was observed by 48 h, with a notable increase in apoptotic cells (*p* < 0.001). At 1000 µg/mL, cell viability dropped dramatically, with a substantial transition to late apoptosis or necrosis.

Collectively, the flow cytometry and viability data indicate that apoptosis is the predominant mode of cell death at concentrations ≥ 500 µg/mL, while the 250 µg/mL concentration primarily maintains membrane integrity and cell viability.

### 2.4. Cell Cycle Arrest Mechanism

Flow cytometry analysis indicated that *J. spicigera* extract induced dose-dependent changes in the cell cycle distribution of LNCaP cells. At 250 µg/mL, a significant accumulation of cells in the G_0_/G_1_ phase was observed, alongside a reduction in the S phase population (*p* < 0.01). This pattern intensified at 500 µg/mL, with increased G_0_/G_1_ arrest, further S phase depletion, and a notable rise in the sub-G_0_/G_1_ fraction, consistent with DNA fragmentation and the onset of apoptosis (*p* < 0.001). At 1000 µg/mL, the sub-G_0_/G_1_ population became dominant, indicating that apoptotic cell death supersedes cytostatic effects at higher concentrations.

## 3. Discussion

**Figure 9:**
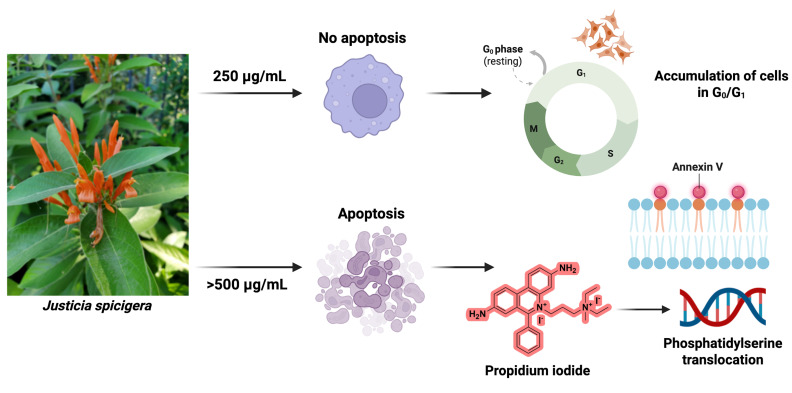
Proposed model of the dose-dependent effect of *J. spicigera* extract on LNCaP prostate cancer cells. At 250 µg/mL, the extract induces G_0_/G_1_ arrest, reducing proliferation without triggering apoptosis. At higher concentrations (≥500 µg/mL), it initiates apoptosis through phosphatidylserine translocation and DNA fragmentation.

This study provides a foundational analysis of the antiproliferative effects of a hydroalcoholic extract of *J. spicigera* on LNCaP prostate cancer cells. Our results demonstrate that the extract modulates cell behavior in a distinct, concentration-dependent manner (Figure 9). At a lower concentration (250 µg/mL), the extract consistently induced G_0_/G_1_ cell cycle arrest without triggering significant apoptosis, indicating an interference with early cell cycle checkpoints. In contrast, higher concentrations (≥500 µg/mL) produced a clear cytotoxic response, characterized by the induction of apoptosis, phosphatidylserine translocation, and DNA fragmentation.

The focus on kaempferitrin as a representative marker is supported by its well-documented pro-apoptotic activity in various cancer models. Furthermore, the traditional consumption of *J. spicigera* as a medicinal tea for cancer-related symptoms in Mexico, particularly among male patients, provides a strong ethnomedical basis for investigating these specific flavonoids. While the major unidentified peaks detected in our LC-MS profile (Figure 1B) may contribute to the overall pharmacological effect, kaempferitrin serves as a validated chemical surrogate to explain the biological potential observed in this androgen-sensitive LNCaP model.

The mechanism behind these dual effects appears to be rooted in the extract’s influence on cell cycle progression. The observed G_0_/G_1_ arrest points to a specific disruption of the molecular machinery governing the cell cycle’s initial phase, likely through interference with regulators like cyclin D1/CDK4 [30,33,34]. Our phytochemical analysis, which putatively identified the flavonoid kaempferitrin, provides a strong chemical basis for this activity. This is consistent with the known function of flavonoids, which characteristically target cell cycle regulators and DNA integrity pathways [25], and have been widely implicated in modulating crucial cancer-related pathways such as PI3K/Akt, p53, and MAPK that directly influence cell cycle progression and survival [35,36].

As the concentration of the extract increases, the sustained pressure on the cell cycle likely pushes the cells toward programmed cell death. This transition from cytostasis to apoptosis aligns with reports of flavonoids and phytosterols modulating apoptotic signaling and mitochondrial function in cancer cells [28,29]. The clear pattern of Annexin V positivity and sub-G_0_/G_1_ accumulation at concentrations ≥ 500 µg/mL strongly suggests that this cytotoxicity is mediated by an activation of programmed cell death pathways. Although the causative agent was not isolated, the putative identification of kaempferitrin further supports this hypothesis, as apoptosis induction has been previously linked to kaempferitrin and its analogs in other cancer models [34,35,36,37,38,39,40,41,42,43,44,45].

Our findings advance the work of Fernández-Pomares et al., who reported that *J. spicigera* extract exerted purely cytostatic effects with no significant reduction in viability (IC_50_ > 4000 µg/mL). By contrast, our results demonstrate that while the extract is indeed cytostatic at 250 µg/mL, it initiates a robust apoptotic response at 500 µg/mL (58% late apoptosis). This discrepancy likely stems from our use of Annexin V/PI flow cytometry, a more sensitive measure of membrane symmetry and programmed death than the Ki-67 and Trypan Blue assays employed in earlier studies [32]. This discrepancy may suggest a higher intrinsic resistance of LNCaP cells or a cell-line-specific sensitivity to the extract’s constituents. However, the mechanism of G_0_/G_1_ arrest is consistent with the actions of other well-known, plant-derived agents against prostate cancer. Curcumin and epigallocatechin-3-gallate (EGCG), for example, have both been reported to inhibit LNCaP cell proliferation by inducing G_0_/G_1_ phase cell cycle arrest [46]. Similarly, resveratrol has also been shown to inhibit LNCaP cell proliferation by inducing cell cycle arrest [47]. The fact that the *J. spicigera* extract shares this mechanism highlights its preliminary potential as a relevant agent for controlling the growth of androgen-sensitive prostate cancer in vitro, though its clinical relevance is strictly contingent upon future safety evaluations in non-malignant tissues.

A significant finding of this study is the therapeutic implication of this dual, concentration-dependent mechanism. The selective induction of cell cycle arrest at lower, non-lethal doses suggests a more controlled, cytostatic profile. This contrasts with many conventional chemotherapeutics that exert immediate and often indiscriminate cytotoxicity, leading to significant side effects. Furthermore, compared to conventional chemotherapeutics that often cause G_2_/M arrest, the preferential G_0_/G_1_ arrest observed with *J. spicigera* may offer a different point of intervention, particularly relevant to hormone-sensitive forms of prostate cancer [48].

While this study provides a strong rationale for the anticancer potential of *J. spicigera*, its limitations define clear directions for future research. The causative agent was not isolated, and the identification of kaempferitrin, while based on accurate mass, remains putative without definitive structural confirmation. Future work should focus on isolating this flavonoid to allow for unambiguous structural elucidation through spectroscopic methods like NMR. The most critical next step is to evaluate the extract’s selectivity by testing its effects on non-malignant prostate epithelial cells, which is essential to determine its therapeutic window and potential for safe application. Furthermore, a deeper mechanistic validation through molecular assays targeting key regulators of the cell cycle (CDKs, Rb, p21) and apoptosis (Bcl-2, Bax) is warranted to delineate the precise pathways involved.

While this study provides a rigorous phenotypic characterization of the dose-dependent cytostatic and pro-apoptotic effects of *J. spicigera*, we acknowledge that the precise molecular mechanisms governing these transitions require further elucidation. The observed G_0_/G_1_ arrest strongly suggests the modulation of key cell cycle regulators, such as the inhibition of Cyclin D1/CDK4 complexes or the induction of p21^WAF1/Cip1^. Similarly, the transition to apoptosis at higher concentrations likely involves the activation of the intrinsic mitochondrial pathway, as evidenced by DNA fragmentation in the Sub-G_0_ population. Future investigations utilizing Western blotting and qPCR are warranted to quantify the expression of these specific regulatory proteins and clarify the molecular signaling hierarchy triggered by the extract.

We acknowledge that the effective concentrations identified in this study (250–500 µg/mL) are high compared to purified compounds. However, this is consistent with the evaluation of crude hydroalcoholic extracts, where bioactive markers, such as kaempferitrin, represent only a minor fraction of the total mass. The use of the LNCaP cell line was strategic, as its androgen-sensitive nature allowed for the characterization of cell cycle modulation in a model reflecting early-stage disease. While this foundational study focuses on the phenotypic response of malignant cells, the absence of a non-malignant prostate cell line (RWPE-1) means the Selectivity Index (SI) remains to be formally established. Future studies are required to confirm if the observed cytostatic-to-cytotoxic transition is tumor-specific.

While this study provides a robust phenotypic characterization of *J. spicigera* extract, several limitations are acknowledged. First, the investigation did not include individual flavonoid-specific assays or molecular target validation (Western blotting for Cyclin/CDK complexes), which are necessary to delineate the precise signaling hierarchy. Second, the current results are derived exclusively from the androgen-sensitive LNCaP model; further testing in AR-independent lines and in vivo models is required to confirm broader applicability and systemic safety. Finally, as the study utilized a crude hydroalcoholic extract, the potential for non-specific interactions remains. Future research will focus on bioguided fractionation and network pharmacology to bridge the gap between individual phytochemicals and their synergistic anticancer mechanisms.

In conclusion, this study demonstrates that a hydroalcoholic extract of *J. spicigera* exerts a potent, dual-action antiproliferative effect on LNCaP prostate cancer cells, acting as a cytostatic agent at low concentrations and an apoptosis-inducing agent at higher concentrations. These findings provide the first detailed evidence supporting the potential use of this traditional Mexican medicinal plant for prostate cancer therapy and establish a strong scientific basis for future studies aimed at isolating its bioactive compounds and evaluating its efficacy and safety in vivo.

## 4. Materials and Methods

### 4.1. Plant Material and Extract Preparation

Fresh leaves and stems of *J. spicigera* were collected from Lencero, Veracruz, México (19° 29′ 27.5″ N, 96° 48′ 53.6″ W) in February 2021. The plant was taxonomically identified by Dr. Edison Fernando Nicolalde-Morejón, a plant taxonomist at Universidad Veracruzana, and a voucher specimen (No. 15655) was deposited at the Herbarium of Instituto de Investigaciones Biológicas, Universidad Veracruzana, for future reference. The plant material was collected in compliance with national and international guidelines on biodiversity.

The collected plant material was washed with distilled water, air-dried at room temperature (25 °C, 50–60% relative humidity) for seven days, and manually cut into small pieces. Hydroalcoholic extraction was performed by macerating 90 g of powdered plant material in 500 mL ethanol:water (50:50) at room temperature for 72 h with occasional stirring. The extract was filtered using Whatman No. 1 filter paper (Cytiva, Marlborough, MA, USA), concentrated under reduced pressure using a rotary evaporator (Büchi R-210, Flawil, Switzerland) at 40 °C, and lyophilized in a freeze dryer (Labconco FreeZone 4.5, Kansas City, MO, USA) to obtain a dry extract. The hydroalcoholic extraction of 980 g of powdered plant material yielded 56 g of dry extract, corresponding to a yield of 5.71%.

### 4.2. Preliminary Phytochemical Characterization

The phytochemical composition of the *J. spicigera* hydroalcoholic extract was analyzed using TLC and LC-MS. Based on literature reports of its significant bioactivity, kaempferitrin was selected as a chemical marker for putative identification, although it was not the most abundant compound in the extract.

TLC was performed using silica gel 60 F254 plates (Merck, Darmstadt, Germany) as the stationary phase. The extract (5 µL) was applied as a spot and developed in a 1-butanol:water:acetic acid (6:3:1) solvent system. Plates were visualized under UV light (254 nm and 365 nm), followed by spraying with 1% aluminum chloride to detect flavonoids.

LC-MS analysis was performed on an Ultimate 3000 liquid chromatograph (Dionex Corp., Sunnyvale, CA, USA) coupled to a micrOTOF-Q II mass spectrometer (Bruker Daltonics, Billerica, MA, USA). Separation was performed on a C18 Hypersil column (5 µm, 250 × 4.6 mm). The mobile phase consisted of 0.01% formic acid in water (Solvent A) and acetonitrile (Solvent B), with a flow rate of 0.3 mL/min. The mass spectrometer was operated with an electrospray ionization (ESI) source in positive ion mode. The key parameters were: capillary voltage, 4500 V; nebulizer pressure, 1.6 Bar; dry gas flow, 12.0 L/min; and dry heater temperature, 200 °C. The mass scan range was set from *m*/*z* 50 to 3000.

### 4.3. Cell Culture and Maintenance

The human prostate cancer cell line LNCaP (ATCC© CRL-1740™) was obtained from ATCC (Manassas, VA, USA) and maintained in RPMI-1640 medium (Gibco, Waltham, MA, USA) supplemented with 10% fetal bovine serum (FBS, Gibco, Waltham, MA, USA), 1% penicillin–streptomycin (Sigma-Aldrich, St. Louis, MO, USA), and 2 mM L-glutamine (Gibco, Waltham, MA, USA). Cells were incubated at 37 °C in a humidified atmosphere with 5% CO_2_ and passaged every 2–3 days upon reaching 70–80% confluence using 0.25% trypsin-EDTA (Gibco, Waltham, MA, USA).

Cell viability was monitored using the trypan blue exclusion assay, and only cultures with ≥95% viability were used for experiments. The absence of Mycoplasma contamination was routinely verified using a PCR-based Mycoplasma detection kit (Lonza, Basel, Switzerland).

For experimental treatments, LNCaP cells were seeded into 96-well, 12-well, or 6-well plates, depending on the assay requirements, and allowed to adhere for 24 h before treatment. Cells were then treated with varying concentrations of *J. spicigera* extract, while 0.1% DMSO served as the vehicle control (negative control). The LNCaP cell line was specifically selected to test the prospective hypothesis that the flavonoid content of *J. spicigera* could interfere with hormone-sensitive proliferation. Rather than relying solely on general ethnopharmacological claims, this experimental framework targets a specific, clinically relevant phenotype of prostate cancer. The choice of a single cell line model (LNCaP) served to define the specific concentration-dependent ‘switch’ from G_0_/G_1_ arrest to apoptosis. We acknowledge that the lack of comparison with a normal prostate cell line is a limitation of this initial mechanistic validation, which we aim to address in follow-up selectivity assays.

### 4.4. MTT Assay for Cell Viability

The MTT assay was used to evaluate the effect of *J. spicigera* extract on LNCaP cell viability. This colorimetric assay measures the reduction of MTT into formazan crystals by mitochondrial dehydrogenases in metabolically active cells, with absorbance intensity correlating with the number of viable cells [49].

LNCaP cells were seeded in 96-well plates at 8 × 10^4^ cells per well in 100 µL of RPMI-1640 medium and allowed to adhere for 24 h at 37 °C in a 5% CO_2_ incubator. After adherence, cells were treated with serial dilutions of J. spicigera extract (ranging from 62.5 to 4000 µg/mL) for 24, 48, and 72 h, with 0.1% DMSO serving as the vehicle control. Each condition was tested in triplicate wells across three independent experiments [50].

Following the incubation period, 10 µL of MTT (M2128, Sigma-Aldrich, St. Louis, MO, USA) solution (5 mg/mL in PBS, Sigma-Aldrich, St. Louis, MO, USA) was added to each well, and plates were incubated for 3 h at 37 °C. The supernatant was removed, and 100 µL of DMSO (Sigma-Aldrich, St. Louis, MO, USA) was added to dissolve the formazan crystals. Absorbance was measured at 570 nm, with background correction at 650 nm, using a microplate reader (Multiskan GO, Thermo Fisher Scientific, Waltham, MA, USA). The assignment of different extract concentrations to the wells of the microplates was performed randomly.

Cell viability was calculated relative to the untreated control using the formula:

$$Cell\;viability\left(\%\right)=\frac{Absorbance\;of\;treated\;cells}{Absorbance\;of\;control\;cells}\times 100$$

### 4.5. Trypan Blue Exclusion Assay

The Trypan Blue exclusion assay was used to assess LNCaP cell viability following treatment with *J. spicigera* extract. This method differentiates between viable and non-viable cells, as intact plasma membranes exclude Trypan Blue, whereas compromised membranes allow dye uptake, making non-viable cells appear blue under a microscope [51]. LNCaP cells were seeded in 12-well plates at 1 × 10^5^ cells per well in 1 mL of RPMI-1640 medium (R8005, Sigma-Aldrich, St. Louis, MO, USA) and allowed to adhere for 24 h at 37 °C in a humidified incubator with 5% CO_2_. After adherence, cells were treated with varying concentrations of *J. spicigera* extract (specifically 62.5, 250, 500, and 1000 µg/mL), with 0.1% DMSO as the vehicle control, 10 µM etoposide (10 µM; Sigma-Aldrich, St. Louis, MO, USA) and 5-fluorouracil (10 µM; Sigma-Aldrich) as the positive controls. Each condition was tested in triplicate wells across three independent experiments [50].

At the end of the incubation period, the culture medium was removed, and cells were trypsinized with 0.25% Trypsin-EDTA (Gibco, Waltham, MA, USA) for 2 min at 37 °C. Trypsinization (T3924, Sigma-Aldrich, St. Louis, MO, USA) was halted by adding an equal volume of complete RPMI-1640 medium (10% FBS), and cells were resuspended in PBS. A 10 µL aliquot of the suspension was mixed with 10 µL Trypan Blue dye (0.4%, Sigma-Aldrich, St. Louis, MO, USA) in a 1:1 ratio, and 10 µL of the mixture was loaded onto a Neubauer hemocytometer for manual counting under a light microscope (Leica DM500, Wetzlar, Germany). Cell viability was calculated using the formula:

$$Viability\left(\%\right)=\frac{Number\;of\;unstained\;cells}{Total\;number\;of\;cells}\times 100$$

### 4.6. Flow Cytometry for Apoptosis (Annexin V/PI Staining)

Apoptosis was assessed using Annexin V/PI staining followed by flow cytometry to distinguish viable, early apoptotic, late apoptotic, and necrotic cells based on phosphatidylserine externalization and membrane integrity [52].

LNCaP cells were seeded in 6-well plates at 2 × 10^5^ cells per well in 2 mL of RPMI-1640 medium and allowed to adhere for 24 h at 37 °C in a humidified incubator with 5% CO_2_. Cells were then treated with varying concentrations of *J. spicigera* extract (specifically 250, 500, and 1000 µg/mL), with 0.1% DMSO as the vehicle control and 10 µM etoposide (Sigma-Aldrich, St. Louis, MO, USA) as the positive control. Each condition was tested in three independent experiments [50].

Following treatment, cells were harvested by trypsinization (0.25% Trypsin-EDTA, Gibco, Waltham, MA, USA) for 2 min at 37 °C, neutralized with RPMI-1640 medium containing 10% FBS, and resuspended in cold PBS. A total of 1 × 10^5^ cells were transferred to fluorescence-activated cell sorting (FACS) tubes, centrifuged at 300× *g* for 5 min, and resuspended in 100 µL of 1× Annexin V binding buffer (BD Biosciences, Franklin Lakes, NJ, USA). Cells were stained with 5 µL of Annexin V-FITC and 5 µL of propidium iodide (PI, 50 µg/mL, BD Biosciences, Franklin Lakes, NJ, USA) and incubated in the dark for 15 min at room temperature.

After incubation, 400 µL of 1× binding buffer was added, and samples were immediately analyzed using a flow cytometer (BD Accuri C6, BD Biosciences, Franklin Lakes, NJ, USA). Data was acquired from at least 10,000 events per sample, and analysis was performed using FlowJo v10 software (BD Biosciences, Franklin Lakes, NJ, USA).

Apoptotic cell populations were classified as: (1) annexin V^−^/PI^−^ for viable cells; (2) annexin V^+^/PI^−^ for early apoptotic cells; (3) annexin V^+^/PI^+^ for late apoptotic cells; and (4) annexin V^−^/PI^+^ for necrotic cells [52].

### 4.7. Cell Cycle Analysis (PI Staining by Flow Cytometry)

To evaluate the effects of *J. spicigera* extract on cell cycle progression in LNCaP cells, PI staining followed by flow cytometry was performed. This method quantifies DNA content in individual cells, enabling the identification of G_0_/G_1_, S, and G_2_/M phases, as well as sub-G_0_/G_1_ populations indicative of apoptotic DNA fragmentation [53]. LNCaP cells were seeded at 2 × 10^5^ cells per well in 6-well plates containing 2 mL of RPMI-1640 medium and incubated at 37 °C with 5% CO_2_ for 24 h to allow adherence. Cells were then treated with varying concentrations of *J. spicigera* extract (specifically 250 and 500 µg/mL), with 0.1% DMSO as the vehicle control, 10 µM etoposide (Sigma-Aldrich, St. Louis, MO, USA) and 10 µM 5-fluorouracil (Sigma-Aldrich) as the positive controls. Each condition was tested in three independent experiments [50,53].

Following treatment, both floating and adherent cells were collected by trypsinization (0.25% Trypsin-EDTA, Gibco, Waltham, MA, USA) and centrifugation at 300× *g* for 5 min at 4 °C. The cell pellet was washed twice with cold phosphate-buffered saline (PBS, pH 7.4), resuspended in 500 µL of ice-cold PBS, and fixed by adding 1 mL of 70% ethanol dropwise while vortexing. Fixed cells were stored at −20 °C for at least 24 h before further processing. On the day of analysis, ethanol-fixed cells were centrifuged at 500× *g* for 5 min, washed twice with PBS, and incubated with RNase A (100 µg/mL, Sigma-Aldrich, St. Louis, MO, USA) at 37 °C for 30 min to eliminate RNA contamination. PI staining was performed by adding 50 µg/mL propidium iodide (Sigma-Aldrich, St. Louis, MO, USA) in PBS containing 0.1% Triton X-100, followed by incubation for 15 min in the dark at room temperature.

Flow cytometry analysis was conducted using a BD FACSCanto II flow cytometer (BD Biosciences, Franklin Lakes, NJ, USA), acquiring 10,000 events per sample on the FL2-A channel (PI fluorescence at 620 nm). The cell cycle distribution was analyzed using FlowJo v10 software (BD Biosciences, Franklin Lakes, NJ, USA) with the Watson pragmatic cell cycle modeling algorithm to determine the percentage of cells in G_0_/G_1_, S, and G_2_/M phases. The sub-G_0_/G_1_ population, indicative of apoptotic cells with fragmented DNA, was also quantified [53].

### 4.8. Statistical Analysis

All experiments were performed in triplicate across three independent experiments, and data are presented as mean ± standard error of the mean (SEM). Statistical significance was assessed using one-way ANOVA followed by Tukey’s post hoc test, with *p* < 0.05 and *p* < 0.001. Flow cytometry data were analyzed using FlowJo v10 software (BD Biosciences, Franklin Lakes, NJ, USA), and cell cycle distribution was determined using the Watson pragmatic cell cycle modeling algorithm. All graphs and plots were generated using GraphPad Prism 10.0 (GraphPad Software, San Diego, CA, USA).

## 5. Conclusions

This study provides the first detailed evidence that a hydroalcoholic extract of *Justicia spicigera* Schltdl. exerts a potent, dose-dependent antiproliferative effect on androgen-sensitive LNCaP prostate cancer cells. The results establish a dual mechanism of action wherein the extract functions as a cytostatic agent at lower concentrations (250 µg/mL) by inducing significant G_0_/G_1_ cell cycle arrest, while transitioning to a pro-apoptotic agent at higher concentrations (≥500 µg/mL). Phytochemical profiling indicates the presence of kaempferitrin, which, based on established literature for *J. spicigera*, represents a plausible candidate for the observed biological effects. However, at this stage, this connection remains a hypothesis, as the potential contributions or synergistic effects of the major unidentified constituents detected in the extract must be confirmed through future isolation and comparative testing. Altogether, these results provide a preliminary scientific rationale for the traditional use of “muicle”. However, the high concentrations required for the pro-apoptotic response emphasize that the study’s significance is currently limited to a foundational phenotypic characterization. Subsequent research must prioritize the determination of the Selectivity Index using normal prostate epithelial cells to establish a true therapeutic window. By selectively targeting cell cycle checkpoints at sub-lethal doses, *J. spicigera* offers a promising template for developing treatments that could delay tumor progression with potentially lower systemic toxicity than conventional chemotherapeutics. Future research should prioritize the isolation and structural elucidation of the active constituents, alongside in vivo studies to confirm their selectivity, safety, and therapeutic efficacy in complex biological systems.

## Data Availability

The raw data supporting the results of this article is publicly available at the following GitHub repository: https://github.com/tusharpawar49/Justicia-spicigera-LNCap-data (accessed on 15 March 2026).
